# Instruments for Assessing Basic Life Support Knowledge in Secondary School Students: A Scoping Review and Expert Panel

**DOI:** 10.1111/josh.70223

**Published:** 2026-08-25

**Authors:** Marcos Mecías‐Calvo, Maria Sobrido‐Prieto, Rubén Navarro‐Patón

**Affiliations:** ^1^ Department of Applied Learning, Faculty of Teacher Training Universidade de Santiago de Compostela (USC) Lugo Spain; ^2^ Department of Health Sciences, Faculty of Nursing and Podiatry of Ferrol Universidad de A Coruña (UDC) A Coruña Spain

**Keywords:** automated external defibrillator, cardiopulmonary resuscitation, questionnaires, school health, secondary education

## Abstract

**Background:**

Schools are essential settings for teaching Basic Life Support (BLS). However, assessment methods for students vary considerably.

**Methods:**

A scoping review was conducted following the PRISMA‐ScR guidelines and the Joanna Briggs Institute methodology. The search included the MEDLINE, CINAHL, PsycINFO, ERIC, Scopus, and Web of Science databases. Studies using questionnaires administered to secondary school students were selected. A panel of experts validated the descriptive synthesis of the data.

**Results:**

Thirty‐six studies were included. Ad hoc questionnaires are the most common and show significant variations in their structure and the dimensions they assess. Detection of cardiac arrest and the use of automated external defibrillators (AEDs) were examined less frequently than cardiopulmonary resuscitation (CPR) techniques. Studies on long‐term knowledge retention are very limited. Most studies focus solely on immediate postintervention assessments.

**Implications for School Health Policy, Practice, and Equity:**

To ensure uniform and equal training in all educational settings, BLS programs in schools must incorporate all elements of the chain of survival, promote active learning techniques, and employ standardized assessment tools.

**Conclusions:**

Standardized and psychometrically validated instruments are needed to improve the assessment of BLS teaching in secondary schools.

## 1 Introduction

Bystander cardiopulmonary resuscitation (CPR) increases survival after out‐of‐hospital cardiac arrest [1]. Basic life support (BLS) training programs improve knowledge, confidence, and willingness to perform CPR and use automated external defibrillators (AEDs), increasing bystander intervention rates [2, 3, 4]. International guidelines emphasize expanding BLS training within the community, particularly among groups most likely to witness cardiac arrest, through coordinated educational, technological, and organizational strategies [5, 6].

BLS training is increasingly implemented in school settings [5, 6]. Schools are recognized as strategic settings for reaching large youth populations and improving knowledge of CPR and AED use [5, 6]. Evidence shows that primary and secondary school students significantly improve their theoretical knowledge after short, age‐appropriate BLS training programs [7, 8]. International initiatives such as *Kids Save Lives* [9] have promoted the integration of CPR into school curricula, highlighting the need for rigorous assessment of students' knowledge and the role of schools in increasing bystander CPR rates [10].

Current questionnaires range greatly in both structure and content [5]. The number of items, response styles, and domains evaluated in ad hoc, modified, and psychometrically validated measures differ [11, 12, 13]. Important measurement properties such as content validity, internal consistency, and test–retest reliability are not systematically reported. This variability limits comparability between studies, making it difficult to draw conclusions or select appropriate instruments for educational and research settings [5]. A thorough analysis of questionnaires and their measurement characteristics is needed to guide future research and instructional initiatives [5, 10].

It is necessary to thoroughly review the questionnaires used to analyze what high school students know about BLS, including the use of CPR and AED [11, 12, 13]. This study aims to identify and map existing instruments, describe their measurement properties, and examine the content domains and structural characteristics assessed in different educational contexts [5]. This review highlights gaps, methodological inconsistencies, and areas for improvement, contributing to the development and selection of more appropriate tools for evaluating school‐based BLS programs.

## 2 Methods

### 2.1 Study Design

A scoping review was conducted according to the Joanna Briggs Institute (JBI) methodology and reported following PRISMA‐ScR guidelines [14]. The study protocol and additional materials are available on the Open Science Framework (OSF) https://doi.org/10.17605/OSF.IO/MRQE3.

### 2.2 Eligibility Criteria

Studies involving secondary school students were included, regardless of the participants' age at enrollment. Studies assessing BLS knowledge (including CPR and AED use) using questionnaires, including recently developed, adapted, or validated instruments were considered. Intervention and nonintervention studies were included, provided the primary objective was knowledge assessment; studies without training were used only for instrument comparison. Quantitative or mixed‐methods studies reporting at least one measurement property, such as content validity (e.g., expert validation), construct validity, internal consistency, or test–retest reliability, were included, while studies lacking information on instrument characteristics (a total of 8 studies during the full‐text screening phase) were excluded. Only articles published in English or Spanish within the last 10 years were considered, in accordance with the implementation of the *Kids Save Lives* initiative [9].

### 2.3 Information Sources and Search Strategy

A systematic search was conducted in MEDLINE (PubMed), CINAHL, PsycINFO, ERIC, Scopus, and Web of Science. The complete search strategies are detailed in Supporting Information Appendix. The reference lists of the included studies were also reviewed.

### 2.4 Study Selection

Two reviewers independently screened studies in three stages (title, abstract, and full text). Prior to formal screening, both reviewers conducted a calibration pilot on a random sample of 50 articles to ensure consistent and homogeneous application of the eligibility criteria. Following this training, the agreement during the formal screening of the 1566 records was 92.0% (1441/1566 concordant decisions). Discrepancies were minimal and resolved by consensus or with the intervention of a third reviewer.

### 2.5 Data Extraction

Two reviewers independently extracted data using a standardized form. The results were compared to ensure consistency and comprehensiveness, and discrepancies were resolved by consensus or by consulting a third reviewer.

### 2.6 Data Extraction Variables

The extracted data included (1) population and educational context (age or age range, sample size, educational level, and country); (2) training characteristics (type of training, duration, and number of sessions), based solely on explicitly reported information; and (3) instrument‐related variables, including questionnaire name, construct assessed, instrument type (ad hoc, adapted, or validated), number of items, response format, reported measurement properties, and assessment time. In addition, the assessed knowledge content was recorded: recognition of cardiac arrest and initial assessment (consciousness and breathing), activation of the emergency system, Basic Life Support sequence, basic principles of CPR maneuvers (frequency and sequence of compressions and ventilations), use of the AED and, when assessed, knowledge retention.

### 2.7 Data Synthesis

The results were organized according to the questionnaire's characteristics, the target population, and the educational context, after synthesizing the data in a descriptive and narrative manner. Correspondence tables were used to identify usage trends, methodological variations, and gaps in the research.

An expert panel was formed to better understand the results and validate the assessment criteria for basic life support knowledge (including the use of CPR and AEDs) among secondary school students. After analyzing the data, the panel determined the most important domains and thematic areas related to knowledge assessment.

An interdisciplinary expert panel comprising five university academics was convened. The panel included experts in evidence synthesis and research methodology, Applied Didactics, school‐based educational interventions, Basic Life Support education, first aid and lifesaving education, and nursing education. The panel met in a structured 90‐min virtual meeting, moderated jointly by two members of the research team. Panel members reviewed the findings of the scoping review and discussed the core content domains and methodological recommendations for evaluating learning outcomes in Basic Life Support education among secondary school students, with consensus reached through open discussion until unanimous agreement was achieved.

## 3 Findings

**FIGURE 1:**
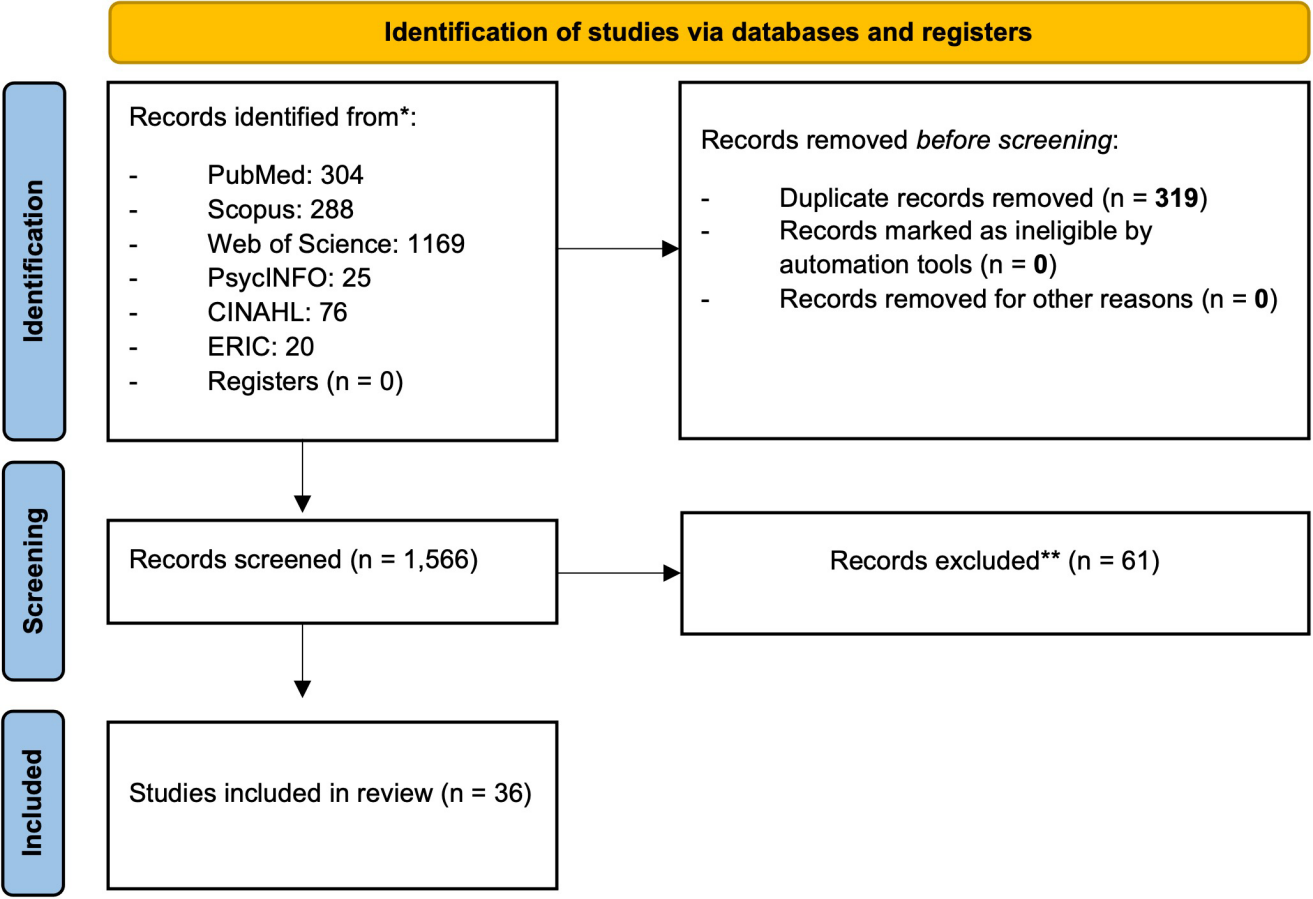
PRISMA 2020 flowchart [15].

Thirty‐six studies were included that assessed high school students' knowledge of basic life support (including the use of CPR and AED) and that were published between 2014 and 2025. The research was carried out in various geographical locations (Figure 1).

The construction, content, and measuring features of the identified instruments varied significantly. Most of the surveys were ad hoc, with a lesser proportion being changed or psychometrically evaluated. The questionnaires created by Borovnik Lesjak et al. [16], Calvo‐Francés et al. [11], and Gutiérrez‐Sánchez et al. [13] showed the strongest psychometric support among the validated instruments. These particular tools offer thorough assessments that methodically address important areas like cardiac arrest identification, the emergency call sequence, CPR technical proficiency, and the functional use of automated external defibrillators (AEDs). Only a small percentage of questionnaires included more than 20 items, with the majority having between 8 and 15.

### 3.1 Characteristics of Included Studies and Instruments

Spain accounted for the majority of the research (*n* = 5), with other European and Asian nations following. Pre–post and quasi‐experimental designs predominated (18/36), with fewer studies being descriptive or instrument validation‐focused (Table 1).

**TABLE 1:** Main characteristics of the included studies and instruments.

References	Year	Design	Country	Sample size	Age range or mean age
Abdelkhalik et al. [17]	2024	Pre–post	Lebanon	401	15–18 years (mean 16.9)
Al Harbi et al. [18]	2018	Cross‐sectional study	Saudi Arabia	1170	15–18 years
Almojarthe et al. [19]	2021	Cross‐sectional study	Saudi Arabia	761	15–20 years (mean 17 ± 1 years)
Baweja et al. [20]	2024	Cross‐sectional study	India	400	13–15 and 16–18 years
Boné et al. [21]	2023	Cross‐sectional study	Portugal	1215	15–16 years
Borovnik Lesjak et al. [16]	2019	Validation	Slovenia	783	12–14 years
Borovnik Lesjak et al. [22]	2022	Pre–post	Slovenia	823	12–14 years
Borovnik Lesjak et al. [2]	2021	Validation	Slovenia	783	12–15 years
Calvo‐Frances et al. [11]	2023	Validation	Spain	259	15–18 years (mean 15.78)
Chilappa et al. [23]	2021	Cross‐sectional study	United States	105	14–19 years
Choi et al. [24]	2015	Quasi‐experimental	South Korea	187 (EG: 119; CG: 68)	15–16 years
Del Rios et al. [25]	2018	Pre–post	United States	71	14–16 years
Fonseca Del Pozo et al. [26]	2016	Quasi‐experimental	Spain	122 (EG: 87; CG: 35)	12–14 years
Gabriel et al. [27]	2019	Pre–post	Nigeria	210	14–19 years
Gradvohl et al. [28]	2023	Pre–post	Hungary	292	12–15 years
Gutiérrez‐Sánchez et al. [13]	2024	Validation	Spain	182	6–16 years
Hamednia et al. [29]	2025	Randomized controlled trial	Iran	200	15–17 years
Huy et al. [30]	2025	Pre–post	Vietnam	806	Not specified
Jabeen et al. [31]	2024	Pre–post	Pakistan	80	15–17 years
Katapadi et al. [32]	2024	Cross‐sectional study	United States	2395	Mean 16.8 years (Unspecified ranges)
Kesici et al. [33]	2021	Quasi‐experimental	Turkey	156	14 years
Losa‐Ballesteros et al. [34]	2020	Observational, prospective, analytical study	Spain	326 (EG: 213; CG: 113)	15–17 years (mean 15.6 years)
Lukas et al. [35]	2016	Prospective observational	Germany	261	10–16 years
Ma et al. [36]	2015	Cross‐sectional study	Hong Kong	383	15–16 years
Navarro‐Patón et al. [37]	2023	Cross‐sectional study	Spain	639	10–17 years
Onan et al. [38]	2019	Quasi‐experimental	Turkey	83	17–18 years
Paglino et al. [39]	2019	Quasi‐experimental	Italy	5146	14–19 years
Pivac et al. [40]	2021	Quasi‐experimental	Slovenia	566	12–15 years
Rovisco‐Branquinho et al. [41]	2021	Pre–post	Portugal	392	7–12 years (mean 8.9 years)
Spartinou et al. [4]	2024	Randomized controlled trial	Greek	408	12–14
Tang et al. [42]	2020	Cross‐sectional study	China	430	15–18 years
Urbina‐Rojas et al. [43]	2022	Validation	Peru	30	11–13 years
Villanueva‐Ordóñez et al. [44]	2019	Quasi‐experimental	Spain	1349	3–15 years
Wetsch et al. [45]	2014	Randomized controlled trial	Germany	342	10–15 years
Wingen et al. [46]	2018	Randomized controlled trial	Germany	424 (EG: 207; CG: 217)	14–18 years
Yeow et al. [47]	2021	Pre–post	Vietnam	118	16 years

Abbreviations: CG: control group; EG: experimental group.

Due to the absence of defined methods for evaluating BLS knowledge in secondary education, the majority of instruments (25/36) were ad hoc. Only a small percentage of questionnaires included more than 20 items; most had eight to fifteen.

### 3.2 BLS Content Knowledge Assessment

CPR maneuvers (compressions and ventilations) were the most frequently assessed content, included in 34 of 36 studies. In contrast, AED use was assessed less frequently (22/36).

Despite their crucial significance in the early activation of the chain of survival, the first steps of the BLS sequence (such as identifying cardiac arrest and the initial assessment of consciousness and breathing) were frequently neglected or only partially addressed (Table 2). The instrument type and the assessed domains are clearly related, according to a cross‐cutting examination of these data. In particular, the scope of ad hoc questionnaires (*n* = 25) was virtually limited to practical CPR techniques (ventilations and compressions). Psychometrically validated tools, on the other hand, consistently provided a more comprehensive assessment, methodically integrating crucial but often disregarded processes including scene safety, cardiac arrest recognition, emergency service activation, and functional AED usage.

**TABLE 2:** BLS content knowledge assessment.

References	Year	Recognition of cardiac arrest	Initial assessment: consciousness and breathing	Activation of emergency services	BLS sequence	CPR maneuvers	AED use	Knowledge retention (when assessed)
Abdelkhalik et al. [17]	2024	No	No	No	Yes	Yes	No	No
Al Harbi et al. [18]	2018	No	Partial/implicit	No	Partial/implicit	Yes	No	No
Almojarthe et al. [19]	2021	No	No	Yes	No	Yes	No	No
Baweja et al. [20]	2024	No	No	Yes	No	No	No	No
Boné et al. [21]	2023	Yes	Yes	Yes	Yes	Yes	Yes	No
Borovnik Lesjak et al. [16]	2019	Yes	Yes	Yes	Yes	Yes	Yes	Yes
Borovnik Lesjak et al. [22]	2022	Yes	Yes	Yes	Yes	Yes	Yes	Yes (5 months and 2 years)
Borovnik Lesjak et al. [2]	2021	Yes	Yes	Yes	Yes	Yes	Yes	Yes
Calvo‐Frances et al. [11]	2023	Yes	Yes	Yes	Yes	Yes	Yes	No
Chilappa et al. [23]	2021	Yes	Yes	Yes	Yes	Yes	Yes	No
Choi et al. [24]	2015	No	No	No	No	Yes	No	Yes (3 months)
Del Rios et al. [25]	2018	Yes	Yes	No	Yes	Yes	Yes	No
Fonseca Del Pozo et al. [26]	2016	Yes	Yes	Yes	Yes	Yes	No	Yes (1 and 8 months)
Gabriel et al. [27]	2019	Yes	Yes	Yes	Yes	Yes	Yes	No
Gradvohl et al. [28]	2023	Yes	Yes	Yes	Yes	Yes	Yes	Yes
Gutiérrez‐Sánchez et al. [13]	2024	Yes	Yes	Yes	Yes	Yes	Yes	No
Hamednia et al. [29]	2025	Yes	Yes	Yes	Yes	Yes	Yes	No
Huy et al. [30]	2025	Yes	Yes	Yes	Yes	Yes	No	No
Jabeen et al. [31]	2024	Yes	Yes	Yes	Yes	Yes	Yes	Yes (3 months)
Katapadi et al. [32]	2024	No	No	No	No	Yes	No	No
Kesici et al. [33]	2021	Yes	Yes	Yes	Yes	Yes	Yes	No
Losa‐Ballesteros et al. [34]	2020	Yes	Yes	Yes	Yes	Yes	No	Yes (2 months)
Lukas et al. [35]	2016	Yes	Yes	Yes	Yes	Yes	No	Yes (3 years, up to 6)
Ma et al. [36]	2015	Yes	Yes	Duda	Yes	Yes	No	No
Navarro‐Patón et al. [37]	2023	Yes	Yes	Yes	Yes	Yes	Yes	No
Onan et al. [38]	2019	Yes	Yes	Yes	Yes	Yes	No	Yes (1 week)
Paglino et al. [39]	2019	Yes	Yes	Yes	Yes	Yes	Yes	Yes (3–6 months)
Pivac et al. [40]	2021	Yes	Yes	Yes	Yes	Yes	Yes	Yes (1–2 months)
Rovisco‐Branquinho et al. [41]	2021	Yes	Yes	Yes	Yes	Yes	No	Yes (6 months)
Spartinou et al. [4]	2024	Yes	Yes	Yes	Yes	Yes	Yes	Yes (6 months)
Tang et al. [42]	2020	Yes	No	Yes	No	Yes	Yes	No
Urbina‐Rojas et al. [43]	2022	Yes	Yes	Yes	Yes	Yes	No	No
Villanueva‐Ordóñez et al. [44]	2019	Yes	Yes	Yes	Yes	Yes	No	No
Wetsch et al. [45]	2014	Yes	Yes	Yes	Yes	Yes	Yes	No (COVID)
Wingen et al. [46]	2018	Yes	Yes	Yes	Yes	Yes	No	Yes (6 months)
Yeow et al. [47]	2021	Yes	Yes	Yes	Yes	Yes	No	Yes (3 months)

### 3.3 Reported Measurement Properties of the Questionnaires

Multiple‐choice questionnaires were the most frequently used format, while dichotomous responses were found mainly in cross‐sectional studies assessing knowledge (Table 3).

**TABLE 3:** Reported measurement properties.

References	Year	Mode of administration	Type of instrument	Number of items (knowledge)	Response format	Assessment timing
Abdelkhalik et al. [17]	2024	Self‐administered online (in the classroom)	Ad hoc	8 items	Dichotomous (true/false)	Pre–post
Al Harbi et al. [18]	2018	In person, self‐administered, in classroom	Ad hoc	5 items	Dichotomous (yes/no) and multiple choice	Cross‐sectional
Almojarthe et al. [19]	2021	In person, self‐administered, in classroom	Ad hoc	5 items	Multiple choice	Cross‐sectional
Baweja et al. [20]	2024	In person, self‐administered, in classroom (paper)	Ad hoc	2 items	Dichotomous (yes/no)	Cross‐sectional
Boné et al. [21]	2023	Self‐administered online	Ad hoc	57 items	Likert/multiple choice (+ some open‐ended)	Cross‐sectional
Borovnik Lesjak et al. [16]	2019	In person, self‐administered, in classroom (paper)	Validated	10 items	Multiple choice	Pre–post
Borovnik Lesjak et al. [22]	2022	In person, self‐administered, in classroom (paper)	Validated	10 items	Multiple choice	Pre–post + follow‐up (5 months, 2 years)
Borovnik Lesjak et al. [2]	2021	In person, self‐administered, in classroom (paper)	Validated	10 items	Multiple choice	Pre–post
Calvo‐Frances et al. [11]	2023	In person, self‐administered, in the classroom	Validated	20 items	Multiple choice	Post
Chilappa et al. [23]	2021	Self‐administered online outside the classroom	Ad hoc	6 items	Multiple choice	Cross‐sectional
Choi et al. [24]	2015	In person, self‐administered, in the classroom	Ad hoc	6 items	Likert	Pre‐post + follow‐up (3 months)
Del Rios et al. [25]	2018	In person, self‐administered, in the classroom	Ad hoc	10 items	Multiple choice	Pre–post
Fonseca Del Pozo et al. [26]	2016	In person, self‐administered, in the classroom	Validated	8 items	Multiple choice	Preintervention, follow‐up at 1 month and at 8 months
Gabriel et al. [27]	2019	In person, self‐administered, in the classroom	AHA Questionnaire	20 items	Multiple choice	Pre–post
Gradvohl et al. [28]	2023	In person, self‐administered, in the classroom	Validated	9 items	Mixed; dichotomous (correct/incorrect, true/false, yes/no), multiple choice (single and multiple selection) and five‐point Likert scale (attitudes).	Pre–post
Gutiérrez‐Sánchez et al. [13]	2024	In person, self‐administered, in classroom (paper)	Validated	12 items	Trichotomous	Pre–post
Hamednia et al. [29]	2025	In person, self‐administered, in the classroom	Validated	9 (from 18 to 26)	Dichotomous	Pre–post
Huy et al. [30]	2025	Self‐administered online (in the classroom)	Ad hoc	35 items	Five‐point Likert scale	Pre–post
Jabeen et al. [31]	2024	In person, self‐administered, in the classroom	Validated	10 items	Multiple choice	Pre–post and follow‐up at 3 months
Katapadi et al. [32]	2024	Online	Ad hoc	9 items	Dichotomous (yes/no), multiple choice and Likert type scales	Cross‐sectional
Kesici et al. [33]	2021	In person, self‐administered, in classroom (paper)	Ad hoc	12 items	Dichotomous and categorical (mainly yes/no; and closed‐choice questions)	Pre–post
Losa‐Ballesteros et al. [34]	2020	In person, in the classroom, on paper	Ad hoc	10 items	Multiple choice	
Lukas et al. [35]	2016	In person, self‐administered, in classroom	Ad hoc	11 items	Multiple choice	Pre–post + follow‐up (6 months)
Ma et al. [36]	2015	In person, self‐administered, in classroom	Ad hoc (tested and with valid content)	10 items	Multiple choice and Likert scale	Cross‐sectional
Navarro‐Patón et al. [37]	2023	Presencial autoadministrada	Ad hoc	14 items	Dichotomous	Cross‐sectional
Onan et al. [38]	2019	In person, self‐administered, in classroom	Ad hoc	15 items	Multiple choice	Pre–post
Paglino et al. [39]	2019	In person, self‐administered, in classroom	Ad hoc	6 items	Dichotomous	Pre–post
Pivac et al. [40]	2021	In person, self‐administered, in classroom	Ad hoc	15 items	Multiple choice (closed)	Delayed pre–post (1–2 weeks)
Rovisco‐Branquinho et al. [41]	2021	In person, self‐administered, in classroom	Ad hoc	10 items	Multiple choice + ordering	Pre–post + follow‐up (6 months)
Spartinou et al. [4]	2024	In person, in classroom	Ad hoc	17 items	Closed (correct/incorrect)	Pre–post + follow‐up 6 months
Tang et al. [42]	2020	In person self‐administered	Ad hoc	13 items	Dichotomous (correct‐incorrect)	Cross‐sectional
Urbina‐Rojas et al. [43]	2022	In person	Validated	8 items	Multiple choice	Cross‐sectional
Villanueva‐Ordóñez et al. [44]	2019	In person, self‐administered, in classroom	Ad hoc	8 items	Multiple choice	Pre–post
Wetsch et al. [45]	2014	In person, self‐administered, in classroom	Ad hoc	12 items	Dichotomous	Pre–post
Wingen et al. [46]	2018	In person, self‐administered, in classroom	Ad hoc	14 items	Multiple choice	Immediate pre–post + follow‐up 6 months
Yeow et al. [47]	2021	In person, self‐administered, in classroom	Ad hoc	14 items	Mixed; dichotomous (correct/incorrect, true/false, yes/no), multiple choice (single and multiple selection) and five‐point Likert scale (attitudes)	Pre–post + follow‐up 3 months

Pre‐ and immediately post intervention assessment was the most common evaluation method. Only 16 of the 36 studies assessed knowledge retention over time, with follow‐up periods generally ranging from 1 to 6 months. In particular, only two studies conducted long‐term follow‐ups exceeding 1 year, whereas medium‐term retention was assessed between one and 6 months (*n* = 13) and short‐term retention was evaluated within a one‐ to 2‐week timeframe (*n* = 1). This presents a significant challenge from an educational planning perspective: school health programs should incorporate mandatory annual or semiannual refresher sessions into the curriculum rather than relying on one‐off interventions, given that most studies confirm that knowledge of basic life support (BLS) deteriorates rapidly within a matter of months.

### 3.4 Expert Panel Results

The expert panel consisted of professionals with experience in BLS, health education, and research methodology. The panelists reviewed a summary of the scoping review findings and identified key domains and criteria for assessing BLS knowledge in secondary school settings.

#### 3.4.1 Key Content Domains

Experts agreed that questionnaires should include at least four key domains: recognition of cardiac arrest, activation of emergency services, BLS sequence, and CPR maneuvers. While these elements are part of a continuous chain of survival, they should be assessed separately to better identify specific knowledge gaps.

#### 3.4.2 Recognition of Cardiac Arrest

Considerable variability was observed among the instruments. Some questionnaires assessed only unconsciousness, while others also included assessment of breathing. Experts emphasized that unconsciousness alone is insufficient to identify cardiac arrest and that an assessment of breathing should be essential. The presence of irregular breathing, such as gasping, was considered relevant depending on the level of training.

#### 3.4.3 Scene Safety

The assessment of the scene safety received inconsistent attention. The panel recommended evaluating it as a broad concept, eliminating overly specialized or technical aspects, and emphasized its importance as a preliminary measure before intervention.

#### 3.4.4 AED Use

Only a portion of the identified questionnaires included questions related to the AEDs' use, and their content varied considerably. Therefore, given the diversity of devices, the experts recommended focusing on functional aspects such as the ability to identify the device, the appropriate time for its use during resuscitation, and the need to follow the instructions provided by the device itself.

#### 3.4.5 Knowledge Retention

Most studies assess knowledge immediately after the training intervention. The expert panel agreed that evaluating knowledge retention is a relevant aspect for assessing the true effectiveness of training programs. However, its implementation can present logistical difficulties in the school setting (loss of follow‐up). Therefore, the panel recommends incorporating knowledge retention assessment when study conditions allow, ensuring the use of comparable instruments across different measurements.

#### 3.4.6 Content Classification by Relevance

The panel proposed classifying the content of the questionnaires into three levels of relevance to facilitate the design of comparable instruments adaptable to different educational contexts: mandatory (present in any questionnaire); recommended (depending on the scope of the training program or the time available); and optional (more specific or advanced aspects).

## 4 Discussion

This study aimed to analyze questionnaires used to assess knowledge of basic life support (BLS), including CPR and the use of an automated external defibrillator (AED), among secondary school students. The results reveal considerable heterogeneity in the design and application of the instruments. Most questionnaires were ad hoc and focused primarily on CPR maneuvers. Assessment was generally limited to immediate pre‐ and postassessments, and few studies examined long‐term retention.

Only a limited number of studies reported validated instruments. Most relied on brief questionnaires (7–30 items) that lacked robust psychometric evidence. These instruments were predominantly self‐administered and ad hoc, with multiple‐choice formats being the most common. The content focused mainly on CPR maneuvers and selected initial steps of the BLS sequence. AED‐related knowledge was assessed less consistently. Assessment approaches were largely based on immediate pre–post designs. Few studies evaluated knowledge retention beyond short follow‐up periods. These findings suggest that BLS programs in schools are often evaluated using brief, nonstandardized tools, which often lack evidence of long‐term reliability and effectiveness.

These findings are consistent with recent international studies. Despite the recognized importance of CPR training in secondary education, many instruments used to evaluate educational interventions lack adequate validation [11] and commonly used questionnaires often have limited psychometric rigor, compromising their reliability and validity [48]. This underscores the need to strengthen the creation and use of more reliable assessment tools.

To facilitate comparability between programs and countries [6], international initiatives such as *Kids Save Lives* [10] emphasize the importance of incorporating BLS instruction in schools, integrating it into curricula and standardized assessment systems. The European Resuscitation Council guidelines call for assessment instruments that are consistent with evidence‐based recommendations and have proven validity and reliability [5]. In this regard, this study contributes to the implementation of international recommendations by identifying both already validated questionnaires and areas of heterogeneity and gaps in content, particularly regarding scene safety, initial recognition, and the use of automated external defibrillators (AEDs), which should be strengthened in future questionnaires.

### 4.1 Implications for School Health Policy, Practice, and Equity

Many questionnaires omit fundamental steps for determining whether to initiate BLS (e.g., recognizing cardiac arrest, initial assessment, or aspects of the AED use). Educational policies should ensure that this content is part of the common curriculum and is assessed uniformly through the integration of standardized evaluation criteria. Furthermore, periodic and longitudinal assessments are proposed to allow for adjustments in training and ensure the maintenance of competencies over time.

Assessment instruments should include clearly defined domains, such as BLS, CPR techniques, identification of cardiac arrest, calling emergency services, functional AED use, and scene safety, which are important components. This could lead to more comprehensive and standardized training methods.

Education should focus on culturally and linguistically appropriate tools, with age‐appropriate response formats. This would help improve the reliability and fairness of assessments across schools and reduce inequalities between schools and countries. Greater standardization of measurement and content would facilitate comparable assessments across different contexts, helping to ensure that all students receive equitable and inclusive basic life support training. Furthermore, policies should promote implementation and evaluation in resource‐limited settings to prevent increased disparities in emergency response capacity.

### 4.2 Limitations

As this was a scoping review, a quantitative quality assessment (e.g., a meta‐analysis) was not performed. Methodological heterogeneity limited comparisons and the generalizability of the results. The review included only published questionnaires, which may have excluded relevant unpublished or emerging tools. Furthermore, the content validity and effectiveness of associated training programs were not examined in depth. Finally, the predominance of studies from specific regions may limit the transferability of the findings to other educational or cultural contexts. Additionally, a potential limitation of this review is language bias. Because the search was restricted to publications in English and Spanish, some relevant studies conducted in other countries and published in other languages may have been omitted.

## 5 Conclusions

This study highlights the considerable variability in questionnaires used to assess BLS knowledge. Most instruments are ad hoc and differ in the number of items, response formats, and content. This limits comparability between studies and makes it difficult to draw conclusions about the effectiveness of educational programs. Assessment focuses primarily on CPR maneuvers. Other components of the chain of survival are assessed less frequently. Furthermore, most studies measure knowledge immediately after training, with little evidence on medium‐ or long‐term retention.

## Funding

Grant PID2022‐143065OA‐I00 funded by MICIU/AEI/10.13039/501100011033 and by ERDF/EU.

## Ethics Statement

Preparation of this paper did not involve primary research or data collection involving human subjects, and therefore, no institutional review board examination or approval was required.

## Conflicts of Interest

The authors declare no conflicts of interest.

## Supporting information


**Data S1:** Full search strategies.

## Data Availability

The data that support the findings of this study are available on request from the corresponding author. The data are not publicly available due to privacy or ethical restrictions.
