# Supplementary material for: Instruments for Assessing Basic Life Support Knowledge in Secondary School Students: A Scoping Review and Expert Panel
Source: J Sch Health. 2026 Aug 25;96(9):e70223. doi: 10.1111/josh.70223 (PMC13505216; doi:10.1111/josh.70223)
Supplement: Supplementary file 1 [file JOSH-96-0-s001.docx]

**Supplementary Appendix. Full search strategies**

**PUBMED**

("Surveys and Questionnaires"[Mesh] OR questionnaire* OR survey*[title] OR assessment* [title] OR appraisal[title] OR measure*[title] OR valida*[title] OR psychosometr* OR

scale*[title])

AND

("Knowledge"[Mesh] OR knowledge[title] OR learn*[title] OR understanding[title] OR Skill*[TITLE])

AND

("Heart Arrest"[Mesh] OR "Cardiopulmonary Resuscitation"[Mesh] OR "Resuscitation"[Mesh] OR "Basic Life Support"[Mesh] OR "basic life support"[title] OR BLS[title] OR CPR[title] OR "cardio pulmonary resuscitation"[title] OR "mouth to mouth resuscitation"[title] OR ventilation[title] OR "rescue breaths"[title] OR "chest compression*"[title] OR "compression only CPR"[title] OR "automated external defibrillator"[title] OR AED[title] OR "first aid"[title])

AND

("Students"[Mesh] OR "Schools"[Mesh] OR "Education"[Mesh] OR school*[title] OR teacher*[title] OR professor*[title] OR pupil*[title] OR student*[title] OR child*[title] OR adolescent*[title] OR youth[title] OR "elementary education"[title] OR "primary education"[title] OR "secondary education"[title] OR "middle school"[title] OR "high school"[title] OR "youth population"[title] OR schoolchild*[title])

Limit: 10 years. Opinion articles were excluded.

**SCOPUS**

(questionnaire* OR survey* OR assessment* OR appraisal* OR valida* OR measure* OR psychosometr* OR Scale*)

AND

(knowledge* OR learning* OR understand* OR Skill*)

AND

TITLE ("basic life support" OR BLS OR CPR OR resuscitat* OR ventilation OR "rescue breaths" OR "chest compression" OR defibrillator* OR AED OR "first aid")

AND

(school* OR teacher* OR professor* OR pupil* OR student* OR child* OR adolescent* OR youth OR Education OR young OR Pediatr*)

Limit: 10 years. Opinion articles were excluded.

Results: 288

**WOS**

(questionnaire* OR survey* OR assessment* OR appraisal* OR valida* OR measure* OR psychosometr* OR Scale*)

AND

(knowledge* OR learning* OR understand* OR OR Skill*)

AND

TITLE ("basic life support" OR BLS OR CPR OR resuscitat* OR ventilation OR "rescue breaths" OR "chest compression" OR defibrillator* OR AED OR "first aid")

AND

(school* OR teacher* OR professor* OR pupil* OR student* OR child* OR adolescent* OR youth OR Education OR young OR Pediatr*)

Limit: 10 years. Opinion articles were excluded.

Results: 1169

**PsycINFO**

TITLE (questionnaire* OR survey* OR assessment* OR appraisal* OR valida* OR measure* OR psychosometr*)

AND

(knowledge* OR learning* OR understand* OR Skill*)

AND

("basic life support" OR BLS OR CPR OR resuscitat* OR ventilation OR "rescue breaths" OR "chest compression" OR defibrillator* OR AED OR "first aid")

AND

(school* OR teacher* OR professor* OR pupil*OR student* OR child*OR adolescent* OR youth OR "elementary education" OR "primary education" OR "secondary education" OR "middle school" OR "high school" OR young OR Pediatr*)

Limit: 10 years. Opinion articles were excluded.

Results: 25

**cinahl**

TITLE (questionnaire* OR survey* OR assessment* OR appraisal* OR valida* OR measure* OR psychosometr*)

AND

(knowledge* OR learning* OR understand*)

AND

("basic life support" OR BLS OR CPR OR resuscitat* OR ventilation OR "rescue breaths" OR "chest compression" OR defibrillator* OR AED OR "first aid")

AND

(school* OR teacher* OR professor* OR pupil*OR student* OR child*OR adolescent* OR youth OR "elementary education" OR "primary education" OR "secondary education" OR "middle school" OR "high school" OR young OR Pediatr*)

Limit: 10 years. Opinion articles were excluded.

Results: 76

**ERIC**

TITLE (questionnaire* OR survey* OR assessment* OR appraisal* OR valida* OR measure* OR psychosometr*)

AND

(knowledge* OR learning* OR understand*)

AND

("basic life support" OR BLS OR CPR OR resuscitat* OR ventilation OR "rescue breaths" OR "chest compression" OR defibrillator* OR AED OR "first aid")

AND

(school* OR teacher* OR professor* OR pupil*OR student* OR child*OR adolescent* OR youth OR "elementary education" OR "primary education" OR "secondary education" OR "middle school" OR "high school" OR young OR Pediatr*)

Limit: 10 years. Opinion articles were excluded.

Results: 20
